# No Evidence of Gouléako and Herbert Virus Infections in Pigs, Côte d’Ivoire and Ghana

**DOI:** 10.3201/eid2112.141840

**Published:** 2015-12

**Authors:** Sandra Junglen, Marco Marklewitz, Florian Zirkel, Robert Wollny, Benjamin Meyer, Hanna Heidemann, Sonja Metzger, Augustina Annan, Dickson Dei, Fabian H. Leendertz, Samuel Oppong, Christian Drosten

**Affiliations:** University of Bonn Medical Center, Bonn, Germany (S. Junglen, M. Marklewitz, F. Zirkel, R. Wollny, B. Meyer, H. Heidemann, C. Drosten);; Robert Koch Institute, Berlin, Germany (S. Metzger, F.H. Leendertz);; Kumasi Centre for Collaborative Research in Tropical Medicine, Kumasi, Ghana (A. Annan);; Ghana Veterinary Services, Kumasi (D. Dei);; Kwame Nkrumah University of Science and Technology, Kumasi (S. Oppong)

**Keywords:** viruses, arbovirus, bunyavirus, insects, Gouléako virus, Herbert virus, vector-borne infections, PCR, arthropods, swine, pigs, mosquitoes, Côte d’Ivoire, Ghana, Africa, South Korea

## Abstract

A recent report suggested that 2 novel bunyaviruses discovered in insects in Côte d’Ivoire caused lethal disease in swine in South Korea. We conducted cell culture studies and tested serum from pigs exposed to mosquitoes in Côte d’Ivoire and Ghana and found no evidence for infection in pigs.

Orthobunyaviruses and phleboviruses are transmitted to animals and humans by blood-feeding arthropods such as mosquitoes, sandflies, and ticks (*1**,**2*). Infection can cause systemic disease, including encephalitis or hemorrhagic fevers. Members of both genera of viruses encode a nonstructural (NS) protein that suppresses the antiviral interferon response of the vertebrate host (*3**,**4*). We recently discovered 2 novel prototypic bunyaviruses in mosquitoes in Côte d’Ivoire (*5**,**6*). Named Gouléako virus (GOLV) and Herbert virus (HEBV), the viruses tentatively define 2 novel bunyavirus-family genera that are in a sister relationship to the genera *Phlebovirus* and *Orthobunyavirus*, respectively. Neither virus encodes NS proteins, nor do the viruses infect vertebrate cells or cause disease in mice that have been intracerebrally inoculated with the viruses (*5**–**7*). Replication of both viruses is blocked at temperatures above 31°C, suggesting that the viruses are unlikely to infect mammals (*8*).

Chung et al. recently reported that, in 2013, GOLV and HEBV caused prevalent and lethal infections in swine in South Korea (*9*). In that study, >500 pigs from 40 farms were tested for both viruses, and viral RNA was detected in up to 79% of diseased and 55% of healthy pigs. Dead pigs carried virus in their lungs and intestines. GOLV was isolated from swine serum in porcine kidney 15 cells. These results suggest the discovery of disease caused by these 2 novel viruses in a major livestock species. Because of the implications of this finding, we attempted verification.

## The Study

**Figure 1:**
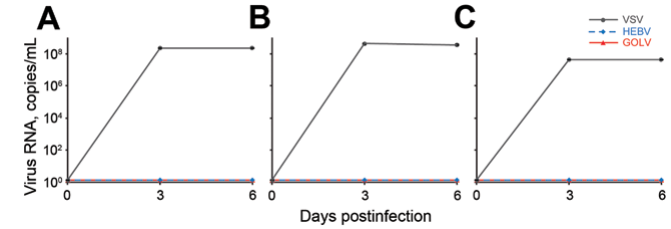
Infection of cells with vesicular stomatitis virus (VSV), Herbert virus (HEBV), and Gouléako virus (GOLV). A) Porcine kidney 15 cells; B) human embryonic kidney cells; C) human hepatocellular 7 cells. Cells were infected at a multiplicity of infection of 1. The number of viral genome copies in cell culture supernatants were measured at 0, 3, and 6 days postinfection by real-time reverse transcription PCR.

We first extended our recent cell culture studies to include porcine kidney 15 and human embryonic kidney 293 cells, which were the type of cells used by Chung et al. (*9*). Human hepatocellular 7 carcinoma cells were also included because they are highly susceptible to virus infection, as are Vero cells and several other cell lines we used in earlier studies (*5**,**6*). Infections with GOLV and HEBV were performed at multiplicities of infection of 1 in doublets in all cell lines. Vesicular stomatitis virus was used as a positive control at multiplicity of infection 1. Cell culture supernatants were analyzed for viral RNA after 0, 3, and 6 days by real-time reverse transcription PCR (RT-PCR) (*5**,**6*). No replication of GOLV and HEBV was detected, whereas vesicular stomatitis virus replicated to high concentrations (Figure 1). Three blind passages on fresh cells failed to yield virus.

Because cell culture experiments may not show the full host range of a specific virus, we tested serum samples collected in 2008 from *Sus scrofa domestica* pigs in Gouléako, the rural village where GOLV and HEBV were first isolated from mosquitoes in Côte d’Ivoire (*5**,**6*). The 28 tested samples represented nearly all the pigs kept in Gouléako at that time, all of which were constantly exposed to mosquitoes. We also tested 108 serum samples collected in 2011 from mosquito-exposed swine in Kumasi, Ghana, where mosquitoes were found to be infected with HEBV (*6*) and GOLV (S. Junglen, unpub. data).

All samples were tested for virus by real-time RT-PCR (*5**,**6*) and tested for antibodies against GOLV and HEBV nucleocapsid proteins by recombinant immunofluorescence assay (*10*). All samples were negative for the viruses (Technical Appendix Table). Technical Appendix Figure 1 shows antigen controls and results from 1 representative swine serum sample.

**Figure 2:**
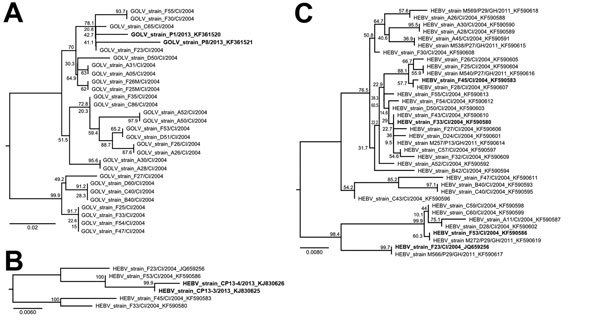
Maximum-likelihood phylogenetic analyses of Gouléako virus (GOLV) and Herbert virus (HEBV) strains from mosquitoes in Côte d’Ivoire, 2004, and Ghana, 2011, and virus strains detected by Chung el al. (*9*) in pigs in South Korea. A) Analysis of the glycoprotein precursor gene of GOLV strains identified in mosquitoes collected in Côte d’Ivoire and Ghana and of strains detected in swine in South Korea. Sequences originating from swine are shown in bold. B) Analysis of the RNA-dependent RNA polymerase gene of HEBV strains from mosquitoes and swine. Sequences originating from swine are shown in bold. C) Analysis of all identified HEBV strains found in mosquitoes. HEBV strains used for phylogenetic analyses in panel B are shown in bold. GOLV strains F25M/CI/2004 and F26M/CI/2004 were found in male mosquitoes. Scale bars indicate nucleotide substitutions per position in the alignment.

To compare the viruses found in pigs in South Korea with viruses found in mosquitoes in Africa, we replicated methods used by Chung et al. (*9*) and amplified a region of the GOLV glycoprotein precursor gene from 27 GOLV strains in mosquitoes (Technical Appendix). Nucleotide sequence distance among mosquito strains was as high as 9.0%. The viruses found in the pigs fell within the genetic diversity of viral strains of GOLV and HEBV and did not constitute phylogenetic outliers (Figure 2, panel A). The analyzed fragment had 6 aa exchanges, but they were insufficient for drawing conclusions about protein function because the fragment did not include domains putatively relevant for receptor binding (Technical Appendix Figure 2).

Small RT-PCR fragments from the RNA-dependent RNA polymerase (RdRp) gene were presented by Chung et al. for HEBV. We performed phylogenetic analyses to compare these swine-derived sequences with sequences from all mosquito-derived viruses from which we could sequence the corresponding genome region (Figure 2, panel B). Comparison of swine-derived sequences with the phylogeny of mosquito-derived HEBV strains, constructed on the basis of the third conserved region of the RdRp (Figure 2, panel C), showed that the strains from South Korea fell within the phylogenetic diversity of HEBV strains identified in West Africa. Technical Appendix Figure 3 shows nucleotide- and amino acid–based alignments.

Our results contrast with those of Chung et al. (*9*) for several possible reasons. First, the viruses infecting swine in South Korea may constitute variants of GOLV and HEBV that can infect vertebrates. The presence of an NSs protein in phleboviruses and orthobunyaviruses provides interferon resistance required to infect vertebrates efficiently (*3**,**4*). Because full genome sequences from swine viruses detected by Chung et al. are not available, we have no information on the presence of NS proteins in these viruses. Furthermore, our detection assays might have failed to detect variant viruses. However, our RT-PCR assays have been shown to detect variant viruses, have been validated for sensitivity (≈100 viral genome copies per mL in liquid specimens), and provide high specificity by probe detection (*5**,**6*). A concern regarding the results of Chung et al. is the use of RT-PCR assays based on SYBR Green (Thermo Fisher Scientific, Lithuania) product detection, which, from our experience, is prone to yield nonspecific results because no probe is used in this assay. Nevertheless, RT-PCR products in Chung et al. have been confirmed by sequencing. Some sequences presented by these researchers contained stop codons in the HEBV RdRp and the GOLV glycoprotein precursor genes, making it unlikely that these sequences represent replicating viruses. Besides technical explanations, these sequences could represent viral genome fragments integrated in genomes of organisms, such as insects, that are eaten by pigs in the region. Integration of RNA virids derived from flaviviruses into the host genome has been described in insects (*11*). Testing food eaten by swine for insect DNA or viral RNA could yield insight. In addition, we may have collected serum when no active virus infections occurred in tested animals. However, past infections would have been shown by antibody tests. Because bunyaviruses from all vertebrate-infecting genera induce antibodies against the nucleoprotein (*12**–**14*), we are confident about our choice of antigen in our assays. Chung et al. presented no serologic results to support virus detections (*9*).

Several technical issues in the study by Chung et al. should be clarified further. First, RNA concentration in tissue, as determined by RT-PCR, did not correlate with the success of probe-based immunohistochemistry in several organ samples (*9*). Second, supernatants from the virus isolate from South Korea showed high cytopathogenic activity in cell culture (10^3^–10^5^ cytopathogenic units/mL) but low levels of concomitant viral RNA by RT-PCR. Because no antigen detection in cells was attempted, the cytopathogenic effect could have been caused by any other virus blindly isolated. One of the most infectious and deadly swine pathogens, the porcine reproductive and respiratory syndrome virus (*15*), was co-detected in lung samples of dead pigs in South Korea (*9*).

The finding of genome fragments of GOLV and HEBV in swine in South Korea needs to be more fully explored. However, with no further independent proof of infection of swine or other vertebrates, HEBV and GOLV should not be considered epizootic pathogens or arboviruses.

**Technical Appendix.** Detailed methods and results of testing of serum samples from pigs for possible infection with Gouléako and Herbert viruses, Côte d’Ivoire, 2008, and Ghana, 2011.
