# Supplementary material for: No Evidence of Gouléako and Herbert Virus Infections in Pigs, Côte d’Ivoire and Ghana
Source: Emerg Infect Dis. 2015 Dec;21(12):2190–3. doi: 10.3201/eid2112.141840 (PMC4672453; doi:10.3201/eid2112.141840)
Supplement: Supplementary file 1 — Technical Appendix. Detailed methods and results of testing of serum samples from pigs for possible infection with Gouléako and Herbert viruses, Côte d’Ivoire, 2008, and Ghana, 2011. [file 14-1840-Techapp-s1.pdf]

Article DOI: http://dx.doi.org/10.3201/eid2112.141840

No Evidence of Gouléako and Herbert Virus Infections in Pigs, Côte d'Ivoire and Ghana

Technical Appendix

Additional Methods and Results

Growth Kinetics

Porcine kidney (PK)-15 cells, human embryonic kidney (HEK)-293 cells, and human hepatocellular carcinoma (HuH)-7 cells were infected with Gouléako virus (GOLV), Herbert virus (HEBV), and vesicular stomatitis virus (VSV) as a positive control at multiplicities of infection (MOI) of 1, as described (*1*,*2*). Cell culture supernatants were analyzed for viral genome copy numbers at 0, 3, and 6 days postinfection by real-time reverse transcription PCR (*1*,*2*).

Amplification of GOLV Glycoprotein Precursor Gene Sequences

RNA was extracted from infected C6/36 cells by using the Viral RNA Kit (QIAGEN, Hilden, Germany) and cDNA synthesis was performed by using SuperScript III (Thermo Fisher Scientific, Lithuania) Glycoprotein precursor gene fragments were amplified by using primers based on strain GOLV/A5/CI/2005 and Platinum Taq polymerase, according to the manufacturer's instructions (Thermo Fisher Scientific, Lithuania). PCR products were analyzed by agarose gel electrophoresis and sequenced by Seqlab (Göttingen, Germany). Sequences were deposited in the GenBank database (National Center for Biotechnology Information, Bethesda, MD, USA) under accession number KT387771–KT387796.

Phylogenetic Analyses

GOLV glycoprotein precursor gene and HEBV RdRp sequences were aligned by using the multiple sequence alignment program MAFFT (http://wiki.hpc.ufl.edu/doc/PhyML); maximum likelihood analyses were inferred by using PhyML (https://code.google.com/p/phyml/) with the HKY85 substitution matrix and 1,000 bootstrap replicates in Geneiuos (Biomatters, Aukland, New Zealand; http://www.geneious.com/).

PCR Screening of Swine Serum Samples

Ethical review and clearances of animal handling procedure were obtained from the Ghana Forestry Commission of the Ministry of Food and Agriculture. RNA was extracted from 15µL of porcine serum samples mixed with 55µL Dulbecco's Phosphate-Buffered Saline by using the QIAamp Viral RNA Mini Kit (QIAGEN, Hilden, Germany). Random cDNA synthesis was performed by using SuperScript III (Thermo Fisher Scientific, Lithuania). Viral genome copies were measured by real-time reverse–transcription PCR, as described previously (*1*,*2*). The Technical Appendix Table shows samples tested and results.

Recombinant Nucleocapsid Immunofluorescence Assay (IFA)

Porcine serum samples were screened for presence of antibodies against the GOLV and HEBV viruses in 1:20 dilutions by rIFA as described (*3*). C-terminally FLAG-tagged full nucleocapsid genes of GOLV or HEBV were amplified from cDNA by using the primers GOLV-N-XbaI-F (5′-GCTCTAGAGCCACCATGGCAACAGTTACTCAGAATGACATTCAG), GOLV-N-FLAG-C-XbaI-R (5′-GCTCTAGATCACTTGTCATCGTCGTCCTTGTAGTCACCAGCTTCCATCAGTTTTCCGGCCGC), HEBV-N-*Bam*HI-F (5′-CGGGATCCGCCACCATGGCTACCAATTTTGAATTCAATGATAAC), and HEBV-N-FLAG-C-SphI-R (5′-ACATGCATGCTCACTTGTCATCGTCGTCCTTGTAGTCACCAGCTTGAGGCCATATTTTGTTGATCAGTG). The amplified genes were then cloned into a pCG1 eukaryotic expression vector.

Plasmids were sequence confirmed. Transfected cells were used in indirect immunofluorescence assays with goat anti-swine IgG-Alexa Fluor 488 conjugate (Sigma, St. Louis, USA) in 1:200 dilution for detection of bound swine serum antibody. A rabbit anti-FLAG antibody and goat anti-rabbit fluorescein-labeled conjugate in 1:200 dilution (Dianova, Hamburg, Germany) were used to confirm expression of viral proteins. A c-terminal flag tag will be expressed only when the upstream viral protein ORF is intact. Cell nuclei were stained with ProLong Gold Antifade Mountant (Thermo Fisher Scientific, Lithuania) with DAPI (4', 6-diamidino-2-phynylindole).

References

1. Marklewitz M, Handrick S, Grasse W, Kurth A, Lukashev A, Drosten C. Gouléako virus isolated from West African mosquitoes constitutes a proposed novel genus in the family Bunyaviridae. J Virol. 2011;85:9227–34. http://dx.doi.org/10.1128/JVI.00230-11

2. Marklewitz M, Zirkel F, Rwego IB, Heidemann H, Trippner P, Kurth A. Discovery of a unique novel clade of mosquito-associated bunyaviruses. J Virol. 2013;87:12850–65. http://dx.doi.org/10.1128/JVI.01862-13

3. Meyer B, Müller MA, Corman VM, Reusken CB, Ritz D, Godeke GJ, et al. Antibodies against MERS coronavirus in dromedary camels, United Arab Emirates, 2003 and 2013. Emerg Infect Dis. 2014;20:552–9 http://dx.doi.org/10.3201/eid2004.131746.

4. Chung HC, Nguyen VG, Goede D, Park CH, Kim AR, Moon HJ, et al. Gouléako and Herbert viruses in pigs, Republic of Korea, 2013. Emerg Infect Dis. 2014;20:2072–5. http://dx.doi.org/10.3201/eid2012.131742

Technical Appendix Table. Porcine serum samples tested for infection with GOLV and HEBV by using real-time reverse transcription PCR and rIFA, in Côte d'Ivoire and Ghana, 2008–2011*

					rIFA		Viral RNA	
Sample ID	Sex	Age, mo†	Origin	Year	GOLV	HEBV	GOLV	HEBV
CI-S 01	Male	12	Gouléako, CI	2008	Neg	Neg	Neg	Neg
CI-S 02	Female	12	Gouléako, CI	2008	Neg	Neg	Neg	Neg
CI-S 03	Female	12	Gouléako, CI	2008	Neg	Neg	Neg	Neg
CI-S 04	Male	11	Gouléako, CI	2008	Neg	Neg	Neg	Neg
CI-S 05	Female	9	Gouléako, CI	2008	Neg	Neg	Neg	Neg
CI-S 06	Female	3	Gouléako, CI	2008	Neg	Neg	Neg	Neg
CI-S 07	Female	3	Gouléako, CI	2008	Neg	Neg	Neg	Neg
CI-S 08	Male	3	Gouléako, CI	2008	Neg	Neg	Neg	Neg
CI-S 09	Female	3	Gouléako, CI	2008	Neg	Neg	Neg	Neg
CI-S 10	Female	6	Gouléako, CI	2008	Neg	Neg	Neg	Neg
CI-S 11	Female	3	Gouléako, CI	2008	Neg	Neg	Neg	Neg
CI-S 12	Female	3	Gouléako, CI	2008	Neg	Neg	Neg	Neg
CI-S 13	Female	12	Gouléako, CI	2008	Neg	Neg	Neg	Neg
CI-S 14	Male	3	Gouléako, CI	2008	Neg	Neg	Neg	Neg
CI-S 15	Female	8	Gouléako, CI	2008	Neg	Neg	Neg	Neg
CI-S 16	Female	4	Gouléako, CI	2008	Neg	Neg	Neg	Neg
CI-S 17	Female	4	Gouléako, CI	2008	Neg	Neg	Neg	Neg
CI-S 18	Female	2	Gouléako, CI	2008	Neg	Neg	Neg	Neg
CI-S 19	Male	2	Gouléako, CI	2008	Neg	Neg	Neg	Neg
CI-S 20	Female	4	Gouléako, CI	2008	Neg	Neg	Neg	Neg
CI-S 21	Male	4	Gouléako, CI	2008	Neg	Neg	Neg	Neg
CI-S 22	Female	2	Gouléako, CI	2008	Neg	Neg	Neg	Neg
CI-S 23	Female	3	Gouléako, CI	2008	Neg	Neg	Neg	Neg
CI-S 24	Female	2	Gouléako, CI	2008	Neg	Neg	Neg	Neg
CI-S 25	Female	3	Gouléako, CI	2008	Neg	Neg	Neg	Neg
CI-S 26	Female	3	Gouléako, CI	2008	Neg	Neg	Neg	Neg
CI-S 27	Female	3	Gouléako, CI	2008	Neg	Neg	Neg	Neg
CI-S 28	Female	5	Gouléako, CI	2008	Neg	Neg	Neg	Neg
GH-S 01	Female	24	Amanfrom, GH	2011	Neg	Neg	Neg	Neg
GH-S 02	Female	6	Amanfrom, GH	2011	Neg	Neg	Neg	Neg
GH-S 04	Male	6	Amanfrom, GH	2011	Neg	Neg	Neg	Neg
GH-S 05	Female	36	Amanfrom, GH	2011	Neg	Neg	Neg	Neg
GH-S 06	Male	6	Amanfrom, GH	2011	Neg	Neg	Neg	Neg
GH-S 07	Male	6	Amanfrom, GH	2011	Neg	Neg	Neg	Neg
GH-S 08	Female	8	Amanfrom, GH	2011	Neg	Neg	Neg	Neg
GH-S 09	Female	8	Amanfrom, GH	2011	Neg	Neg	Neg	Neg
GH-S 10	Female	7	Amanfrom, GH	2011	Neg	Neg	Neg	Neg
GH-S 11	Female	7	Amanfrom, GH	2011	Neg	Neg	Neg	Neg
GH-S 12	Female	1.5	Sokoban New Town, GH	2011	Neg	Neg	Neg	Neg
GH-S 13	Male	6	Dompoase, GH	2011	Neg	Neg	Neg	Neg
GH-S 14	Male	8	Dompoase, GH	2011	Neg	Neg	Neg	Neg

		Age,			rIFA		Viral RNA	
Sample ID	Sex	mo†	Origin	Year	GOLV	HEBV	GOLV	HEBV
GH-S 15	Male	6	Dompoase, GH	2011	Neg	Neg	Neg	Neg
GH-S 16	Female	1.5	Sokoban New Town, GH	2011	Neg	Neg	Neg	Neg
GH-S 17	Female	6	Sokoban New Town, GH	2011	Neg	Neg	Neg	Neg
GH-S 18	Male	1.5	Sokoban New Town, GH	2011	Neg	Neg	Neg	Neg
GH-S 19	Male	6	Sokoban New Town, GH	2011	Neg	Neg	Neg	Neg
GH-S 20	Male	6	Sokoban New Town, GH	2011	Neg	Neg	Neg	Neg
GH-S 21	Female	8	Dompoase, GH	2011	Neg	Neg	Neg	Neg
GH-S 22	Male	6	Dompoase, GH	2011	Neg	Neg	Neg	Neg
GH-S 23	Male	6	Dompoase, GH	2011	Neg	Neg	Neg	Neg
GH-S 24	Male	6	Dompoase, GH	2011	Neg	Neg	Neg	Neg
GH-S 25	Female	6	Dompoase, GH	2011	Neg	Neg	Neg	Neg
GH-S 26	Male	8	Dompoase, GH	2011	Neg	Neg	Neg	Neg
GH-S 27	Female	6	Dompoase, GH	2011	Neg	Neg	Neg	Neg
GH-S 28	Female	8	Dompoase, GH	2011	Neg	Neg	Neg	Neg
GH-S 31	Male	5	Dompoase, GH	2011	Neg	Neg	Neg	Neg
GH-S 32	Male	5	Dompoase, GH	2011	Neg	Neg	Neg	Neg
GH-S 33	Female	5	Akropong, GH	2011	Neg	Neg	Neg	Neg
GH-S 34	Male	5	Akropong, GH	2011	Neg	Neg	Neg	Neg
GH-S 35	Male	5	Akropong, GH	2011	Neg	Neg	Neg	Neg
GH-S 36	Male	5	Akropong, GH	2011	Neg	Neg	Neg	Neg
GH-S 37	Female	5	Akropong, GH	2011	Neg	Neg	Neg	Neg
GH-S 38	Female	7	Akropong, GH	2011	Neg	Neg	Neg	Neg
GH-S 39	Female	7	Akropong, GH	2011	Neg	Neg	Neg	Neg
GH-S 40	Male	6	Akropong, GH	2011	Neg	Neg	Neg	Neg
GH-S 41	Male	6	Akropong 2, GH	2011	Neg	Neg	Neg	Neg
GH-S 42	Male	6	Akropong 2, GH	2011	Neg	Neg	Neg	Neg
GH-S 43	Male	6	Akropong, GH	2011	Neg	Neg	Neg	Neg
GH-S 44	Female	6	Akropong, GH	2011	Neg	Neg	Neg	Neg
GH-S 45	Female	6	Akropong, GH	2011	Neg	Neg	Neg	Neg
GH-S 46	Male	5	Akropong, GH	2011	Neg	Neg	Neg	Neg
GH-S 47	Female	5	Akropong, GH	2011	Neg	Neg	Neg	Neg
GH-S 48	Male	5	Akropong, GH	2011	Neg	Neg	Neg	Neg
GH-S 49	Female	5	Akropong, GH	2011	Neg	Neg	Neg	Neg
GH-S 50	Female	6	Akropong 2, GH	2011	Neg	Neg	Neg	Neg
GH-S 51	Female	6	Akropong 2, GH	2011	Neg	Neg	Neg	Neg
GH-S 52	Male	6	Akropong 2, GH	2011	Neg	Neg	Neg	Neg
GH-S 53	Male	7	Akropong 2, GH	2011	Neg	Neg	Neg	Neg
GH-S 54	Male	7	Akropong 2, GH	2011	Neg	Neg	Neg	Neg
GH-S 55	Female	7	Akropong 2, GH	2011	Neg	Neg	Neg	Neg
GH-S 56	Female	7	Akropong 2, GH	2011	Neg	Neg	Neg	Neg
GH-S 57	Male	7	Akropong 2, GH	2011	Neg	Neg	Neg	Neg
GH-S 58	Male	5	Essienimpong, GH	2011	Neg	Neg	Neg	Neg
GH-S 59	Female	6	Essienimpong, GH	2011	Neg	Neg	Neg	Neg
GH-S 60	Male	5	Essienimpong, GH	2011	Neg	Neg	Neg	Neg
GH-S 61	Male	5	Essienimpong, GH	2011	Neg	Neg	Neg	Neg
GH-S 62	Male	4	Akropong 2, GH	2011	Neg	Neg	Neg	Neg
GH-S 63	Female	4	Akropong 2, GH	2011	Neg	Neg	Neg	Neg
GH-S 64	Male	4	Akropong 2, GH	2011	Neg	Neg	Neg	Neg
GH-S 65	Female	4	Akropong 2, GH	2011	Neg	Neg	Neg	Neg
GH-S 66	Female	4	Akropong 2, GH	2011	Neg	Neg	Neg	Neg
GH-S 67	Male	5	Essienimpong, GH	2011	Neg	Neg	Neg	Neg
GH-S 68	Female	5	Essienimpong, GH	2011	Neg	Neg	Neg	Neg
GH-S 69	Female	5	Essienimpong, GH	2011	Neg	Neg	Neg	Neg
GH-S 70	Female	5	Essienimpong, GH	2011	Neg	Neg	Neg	Neg
GH-S 71	Female	6	Essienimpong, GH	2011	Neg	Neg	Neg	Neg
GH-S 72	Female	6	Essienimpong, GH	2011	Neg	Neg	Neg	Neg
GH-S 73	Female	8	Onwe, GH	2011	Neg	Neg	Neg	Neg
GH-S 74	Female	8	Onwe, GH	2011	Neg	Neg	Neg	Neg
GH-S 75	Female	8	Onwe, GH	2011	Neg	Neg	Neg	Neg
GH-S 76	Male	8	Onwe, GH	2011	Neg	Neg	Neg	Neg
GH-S 77	Male	8	Onwe, GH	2011	Neg	Neg	Neg	Neg
GH-S 78	Female	5	Onwe, GH	2011	Neg	Neg	Neg	Neg
GH-S 79	Male	8	Onwe, GH	2011	Neg	Neg	Neg	Neg
GH-S 80	Female	8	Onwe, GH	2011	Neg	Neg	Neg	Neg
GH-S 81	Female	5	Onwe, GH	2011	Neg	Neg	Neg	Neg
GH-S 82	Female	5	Onwe, GH	2011	Neg	Neg	Neg	Neg
GH-S 83	Female	7	Ejisu Krapa, GH	2011	Neg	Neg	Neg	Neg
GH-S 84	Female	7	Ejisu Krapa, GH	2011	Neg	Neg	Neg	Neg
GH-S 85	Female	7	Ejisu Krapa, GH	2011	Neg	Neg	Neg	Neg
GH-S 86	Female	7	Ejisu Krapa, GH	2011	Neg	Neg	Neg	Neg
GH-S 87	Male	7	Ejisu Krapa, GH	2011	Neg	Neg	Neg	Neg
GH-S 89	Female	5	Ejisu Krapa, GH	2011	Neg	Neg	Neg	Neg
GH-S 90	Female	5	Ejisu Krapa, GH	2011	Neg	Neg	Neg	Neg

Sample ID	Sex	Age, mo†	Origin	rIFA			Viral RNA	
				Year	GOLV	HEBV	GOLV	HEBV
GH-S 91	Male	4	Ejisu Krapa, GH	2011	Neg	Neg	Neg	Neg
GH-S 92	Female	5	Ejisu Krapa, GH	2011	Neg	Neg	Neg	Neg
GH-S 93	Female	12	Abattoir, GH	2011	Neg	Neg	Neg	Neg
GH-S 94	Female	12	Abattoir, GH	2011	Neg	Neg	Neg	Neg
GH-S 95	Female	12	Abattoir, GH	2011	Neg	Neg	Neg	Neg
GH-S 96	Male	12	Abattoir, GH	2011	Neg	Neg	Neg	Neg
GH-S 97	Male	12	Abattoir, GH	2011	Neg	Neg	Neg	Neg
GH-S 98	Female	12	Abattoir, GH	2011	Neg	Neg	Neg	Neg
GH-S 99	Male	12	Abattoir, GH	2011	Neg	Neg	Neg	Neg
GH-S 100	Female	12	Abattoir, GH	2011	Neg	Neg	Neg	Neg
GH-S 101	Female	12	Abattoir, GH	2011	Neg	Neg	Neg	Neg
GH-S 102	Male	12	Abattoir, GH	2011	Neg	Neg	Neg	Neg
GH-S 103	Female	12	Abattoir, GH	2011	Neg	Neg	Neg	Neg
GH-S 104	Female	5	Abattoir, GH	2011	Neg	Neg	Neg	Neg
GH-S 105	Female	12	Abattoir, GH	2011	Neg	Neg	Neg	Neg
GH-S 106	Female	4	Abattoir, GH	2011	Neg	Neg	Neg	Neg
GH-S 107	Male	12	Abattoir, GH	2011	Neg	Neg	Neg	Neg
GH-S 108	Female	12	Abattoir, GH	2011	Neg	Neg	Neg	Neg

*CI, Côte d′Ivoire; GH, Ghana; GOLV, Gouléako virus; HEBV, Herbert virus; Neg, Negative results.
†Age is age of pig from which serum sample was collected.

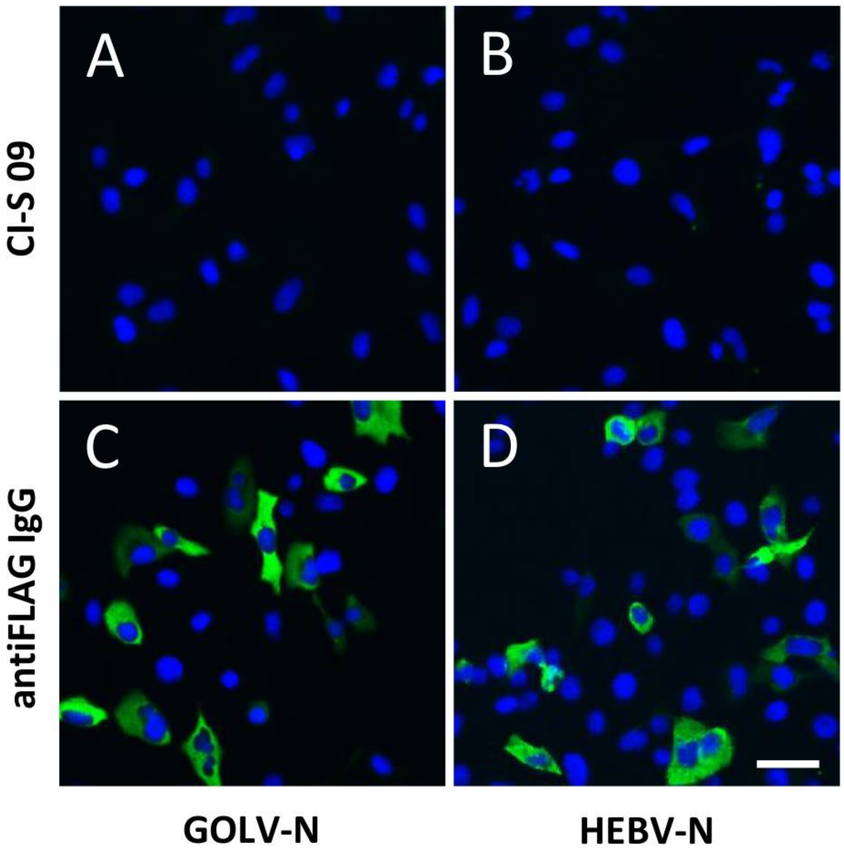

Technical Appendix Figure 1. Immunofluorescence patterns for antibodies against Gouléako virus (GOLV) and Herbert virus (HEBV) in serum samples from swine, Côte d′Ivoire (CI), 2008, and Ghana, 2011. Figure shows representative results from 1 pig (labeled CI-S 09) from which serum was tested against overexpressed recombinant nucleocapsid protein of A) GOLV and B) HEBV in VeroB4 cells. Anti-FLAG IgG antibodies were used to control for overexpression of C) GOLV-nucleocapsid (N) and D) HEBV-N. Scale bar indicates 20 μm. All photographs were taken at equivalent exposure settings.

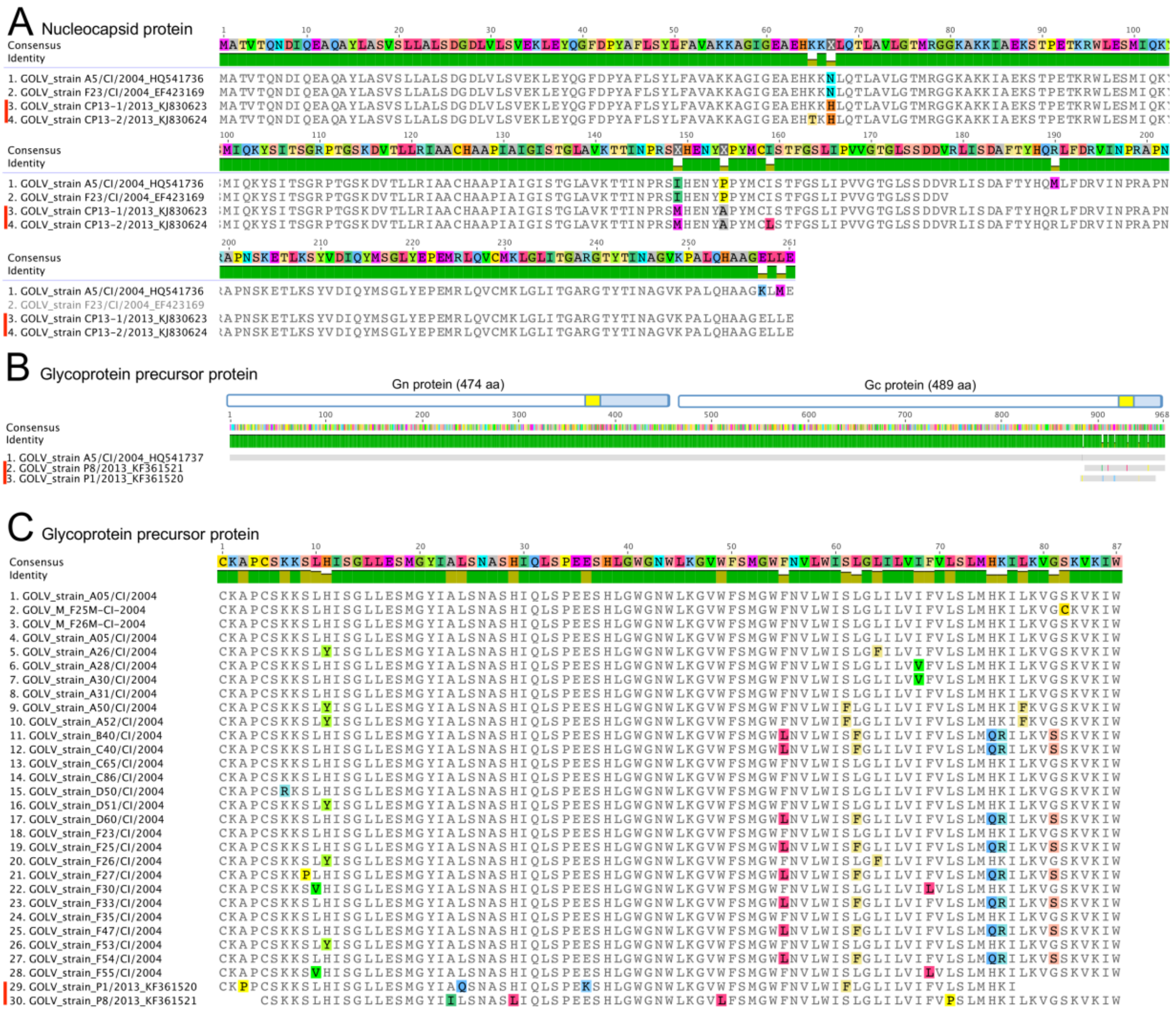

Technical Appendix Figure 2. Alignment of Gouléako virus (GOLV) strains from mosquitoes originating from Côte d'Ivoire and Ghana and swine sampled in Korea. A) Alignment of the GOLV nucleocapsid proteins; B) alignment of complete GOLV glycoprotein precursor protein from mosquito and of protein fragments identified in swine. Schematic overview of encoded proteins is shown in boxes. Transmembrane domains are marked in yellow. Protein domains located outside virions are shown in dark blue, and those located inside virions are in light blue. C) Alignment of Gc proteins originating from mosquitoes and swine. A red line (at consensus identity 29 and 30) marks sequences from swine published by Chung et al. in 2014 (*4*).

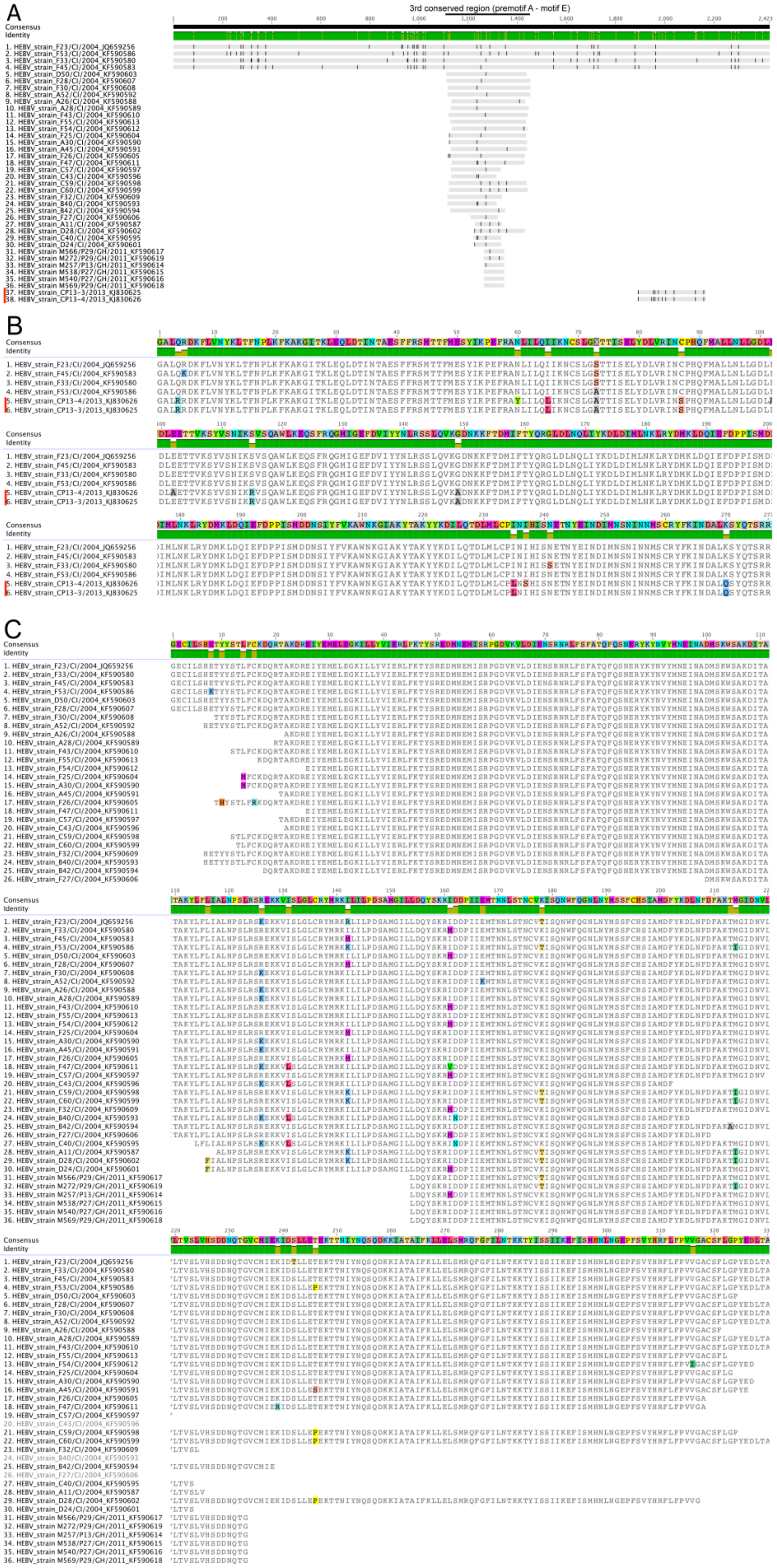
A
3rd conserved region (premotif A - motif E)
Consensus
Identity
B
C

Technical Appendix Figure 3. Alignment of Herbert virus (HEBV) strains from mosquitoes and pigs. A) Overview of location of amplified RNA-dependent RNA polymerase (RdRp) protein sequences available from mosquitoes and swine. Sequences detected in swine in South Korea are indicated by a red line. B) Alignment of RdRp sequences from mosquitoes and swine. Amino acid changes are colored. Sequences detected in swine in South Korea are indicated by a red line. C) Alignment of HEBV protein sequences of the third conserved region of the RdRp identified in mosquitoes from Côte d'Ivoire and Ghana.
